# Autophagic regulation of CD8^+^ T cell metabolic reprogramming defines acute and latent phases of cytomegalovirus infection *in vivo*

**DOI:** 10.3389/fcimb.2025.1673229

**Published:** 2025-12-15

**Authors:** Hui Liu, Xiaobing Liu, Wai Ho Oscar Yeung, Jiang Liu, Kevin Tak Pan Ng, Kwan Man

**Affiliations:** 1 Department of Surgery, School of Clinical Medicine, HKU-SZH & LKS Faculty of Medicine, The University of Hong Kong, Hong Kong, Hong Kong SAR, China; 2 Department of Liver Surgery & Shanghai Institute of Transplantation, Renji Hospital, Shanghai Jiao Tong University School of Medicine, Shanghai, China

**Keywords:** cytomegalovirus, CD8^+^ T cells, autophagic dynamics, metabolism, sprague-dawley rats

## Abstract

Understanding the immunoregulatory mechanism during cytomegalovirus (CMV) infection may help to combat CMV reactivation in immunocompromised or immunosuppressed individuals. Here we developed a CMV infection model in immunocompetent Sprague Dawley (SD) rats with Priscott strain and explored the cross-talk between autophagic dynamics and metabolism alterations in CD8^+^ T cells post infection. We previously found that primary CMV infection induced a remarkable increase of CD8^+^ T cells which reached the peak around week 3 and returned to pre-inoculation status since week 6 post viral infection. In this study, our results demonstrated that the autophagic activity of CD8^+^ T cells was augmented at week 3 while decreased at week 6, which was closely associated with the up- (week 3 and 4) or down-regulation (since week 6) of metabolic markers ENTPD1 and SLC27A2. Furthermore, the *in vitro* study showed that the levels of these metabolic markers in rat splenocytes were modulated by autophagy inhibitors and enhancers. Our study indicated that the dynamic alterations of autophagy exerted a critical role in regulating the metabolic adaptation of CD8^+^ T cells during CMV infection process, and provides an ideal animal model for further research on the pathological mechanisms based on CMV latency.

## 1 Introduction

T-cell responses are pivotal in cytomegalovirus (CMV) control, mediating viral clearance during acute infection, sustaining latency, and suppressing reactivation in immunocompromised individuals, including transplant recipients (Casazza et al., 2006; Castón et al., 2016; Klenerman and Oxenius, 2016). Furthermore, CMV latency-associated T lymphocytes have been proposed as key biomarkers of adverse outcomes following kidney transplantation (Pickering et al., 2021). Nonetheless, the mechanisms governing T-cell responses throughout CMV infection remain incompletely elucidated.

Emerging evidence positions autophagy as a central regulator of T-cell effector function, survival, and metabolic reprogramming in antiviral immunity (Xu et al., 2014; Loi et al., 2016), with particular significance for CD8^+^ T-cell dynamics during CMV infection. Moreover, T-cell metabolism has garnered attention as a therapeutic target to rejuvenate antigen-specific responses in oncology and chronic viral infections (Chang and Pearce, 2016; Fisicaro et al., 2017). However, the regulatory mechanism of T cell metabolism by autophagy during CMV infection still needs to be clarified.

Rat cytomegalovirus CMV (RCMV)-rat system has proven to be very useful in exploring CMV pathogenesis (Bruggeman et al., 1983, 1985). To date, a broad range of rat strains, such as Brown Norway, Lewis, F344 was reported as existing RCMV models. Meanwhile, several strains of RCMV including Maastricht strain (Vink et al., 2000), Priscott strain (Ettinger et al., 2012), and ALL-03 strain (Loh et al., 2003) were identified, among which Maastricht strain was most extensively employed to study antiviral therapy, endovascular infection as well as allograft rejection in solid organ transplantation (Span et al., 1992; Stals et al., 1994; Lemström et al., 1997). Nevertheless, the immunological alteration in response to CMV infection in immunocompetent rats remains poorly defined. To rigorously examine the interplay between T-cell autophagy and metabolic adaptation after CMV inoculation, we developed a rat CMV infection model in immunocompetent Sprague Dawley (SD) rats employing the commercially available Priscott strain.

Here we found CMV inoculation induced cellular immune responses in immuno-competent SD rats comparable with human CMV. And organ latency in liver was developed around 6 weeks after infection. Unexpectedly, we identified that in both acute and latent phases of CMV infection, autophagy modulated the metabolic levels of T cells, especially CD8^+^ T cells to adapt to the stress incurred by viruses. By linking autophagy-associated T-cell metabolic states to the context of CMV infection, our findings may deepen the understanding of T-cell regulation during CMV persistence. Moreover, our RCMV model offers a novel platform for exploring CMV reactivation in clinical scenarios such as liver transplantation.

## 2 Materials and methods

### 2.1 Rat CMV (RCMV) preparation

RCMV (Priscott strain) was purchased from American Type Culture Collection (ATCC, Manassas, VA, USA) and propagated in Rat-2 cells (rat embryonic fibroblast cell line, ATCC) grown in DMEM containing 10% FBS, penicillin (50 unit/ml) and streptomycin (50ug/ml). Rat-2 cells in monolayer at 80% confluence were infected with RCMV at a multiplicity of infection of 0.1. When 100% of the cells exhibited cytopathic effects, the supernatant containing virus was collected and stored at -80 °C. Viral titers were determined via the TCID50 method, based on the appearance of cytopathic effects by serial 10-fold dilutions and expressed as units of 50% tissue culture infective dose (TCID50) assay. The Priscott strain of CMV infection model proposed in this paper is well defined and accepted in SD rats (Zhou et al., 2000).

### 2.2 Rats and virus inoculation

Male SD rats (80~100g) were purchased from the Laboratory Animal Unit, The University of Hong Kong. All animals received humane care according to the criteria outlined in *Guide for the Care and Use of Laboratory Animals* (National Institutes Health publication 86-23, 1985 revision). The experimental protocol was approved by the Committee on the Use of Live Animals in Teaching and Research (CULATR), The University of Hong Kong. All procedures performed in studies involving animals were in accordance with the ethical standards of the institution or practice at which the studies were conducted.

The rats were randomly grouped to receive intraperitoneal injection of either 1ml RCMV (7.9x10^7 TCID50/ml) or mock medium (control). At week 1, 2, 3, 4, 6, 8 (n=6 for each time point, 3 for CMV and 3 for mock medium) post inoculation, the salivary gland, liver and spleen tissues were collected.

### 2.3 Isolation of splenocytes from rats

Isolation of splenocytes from rat spleen was performed according to the protocol described previously with minor modification (Weiss et al., 2008). The isolated splenocytes were cultured in RPMI 1640 containing 10% FBS, penicillin (50 unit/ml), streptomycin (50ug/ml) and stimulated overnight by Rapamycin (#tlrl-rap, InvivoGen, San Diego, CA, USA), Chloroquine diphosphate salt (#C6628-25g, Sigma-Aldrich, St Louis, MO, USA) and Bafilomycin A1 (#1829-50ug, Bio Vision, Mountain View, CA) respectively.

### 2.4 Reverse transcription – quantitative PCR

Total RNA was extracted from rat tissues and splenocytes using Trizol reagent (#15596026, Invitrogen, Carlsbad, CA, USA) or Illustra RNAspin mini kit (#25-0500-72, GE Healthcare) respectively. Then complementary DNA (cDNA) was synthesized with 1μg of total RNA using High Capacity cDNA Reverse Transcription Kit (#43-688-14, Applied Biosystems, Carlsbad, CA, USA). Quantitative real-time PCR was performed with Fast Start SYBR Green Master Mix (#04913914001, Roche Diagnostic systems, Branchburg, NJ, USA). Gene expression was quantified with △△Ct method and normalized to the control (18S).

Total celluar DNA was extracted from salivary and liver using QIAamp DNA mini kit. PCR was performed with Fast Start SYBR Green Master Mix in a total volume of 20 µl containing 0.1 µg of DNA. The sequences of primers were listed in **Table 1** and the PCR conditions were as following: 2 min at 60 °C, 10 min at 95 °C followed by 60 cycles of 95 °C for 15s and 60 °C for 1min. Plots of log(δRn) vs. cycle number were analyzed for the presence of CMV load in organs.

**Table 1:** Primers used for qRT-PCR.

Primers	Forward sequence (5’ to 3’)	Reverse sequence (5’ to 3’)	Annealing temperature	Amplicon size
RCMV IE1/2(DNA)	CCCCGTGACTGCTAAGATCA	CTCGATGTTGGGGTCTCGTC	60	160
Rat Atg 7	TGCAGGGAGCAAGAGATGTG	GGATGACTCAGCCAGCCTTT	60	179
Rat Beclin 1	ATATCTGGCACAGCGGACAA	CCCCAGGCAGCATTGATTTC	60	100
Rat ENTPD1	TGCCCCTTATGGAAGATATAAAGGA	TCCAGCACAATCCCATACTTAACA	60	169
Rat SLC27A2	GTGGTTGGGGCTACATTTGC	GTACCGAAGCAGTTCACCGA	60	114
Rat PD1	TCCTGGAGACCCCAACAAGA	CAGCTTCTCTGGCCTCTGAC	60	171
Rat TIM3	GAACCAGCCAAAGTCATCCCA	TTTCATCGGCCCATGTGGAA	60	157

### 2.5 Flow cytometry

Flow cytometry was carried out with a modified protocol described previously (Ling et al., 2014). The splenocytes were freshly isolated. CMV antigen pool was prepared by heat-inactivating virus (1 h at 56 °C) collected from supernatants of CMV-infected fibroblasts (final concentration of virus was 10^8^p.f.u./ml) (Zhu et al., 2001). Autophagy in CD8^+^ T cells was detected by multicolor analysis after incubation with a dye selectively labeling autophagic vacuoles (Autophagy detection kit #ab139484, Abcam, Cambridge, MA, USA), Percp-CD3 (#46-0030-82, eBioscience, San Diego, CA, USA) and APC-CD8 (#200610, Biolegend, San Diego, CA, USA). All the data was analyzed with FlowJo (Treestar, San Carlos, CA, USA).

### 2.6 Transmission electron microscopy

CD8^+^ T cells were isolated from splenocytes using EasySep™ rat CD8^+^ T cell isolation kit (#19643, Stemcell Technologies, Vancouver, Canada) and immediately fixed in 2.5% glutaraldehyde in sodium carcodylate hydrochloride buffer for 2 hours at 4 °C. Then ultrathin sections were prepared and examined under a transmission electron microscope (Philips CM 100, Philips Industries, Eindhoven, The Netherlands) (Man et al., 2010).

### 2.7 Immunofluorescence staining

The immunofluorescent double staining of CD8 (#200610, Biolegend) and SLC27A2 (#14048-1-AP, Proteintech, Chicago, IL, USA); on formalin-fixed paraffin sections was performed as described previously (Yeung et al., 2015). For CD8 and LC3B (#2775, Cell signaling) staining on rat splenocytes, the cells were cytospinned and fixed with 10% neutral formalin and methonal. The primary antibodies were incubated overnight at 4 °C with the dilution of 1:2000. Wash 3 times by PBS. Alexa Fluor^®^ 488 and Alexa Fluor^®^ 594 (dilution of 1:1000) were used as the secondary antibodies (Invitrogen) for 30 mins at 37 °C. Wash 3 times by PBS and seal the slides using mounting medium with DAPI. medium Images were captured with a confocal microscope (Zeiss, LSM710). We selected twenty double positive cells with complete cell morphology randomly in each slide, quantified the green fluorescence intensity of each cell with Image J and calculated the average value.

### 2.8 Statistics

Data were expressed as mean values with standard error mean. Unpaired *student’s t* test was performed to determine the probability of statistical significance and *p<0.05* was defined as significant. All the analyses were performed using GraphPad Prism 8 (GraphPad Software Inc, La Jolla, CA, USA) or SPSS 18.0 (SPSS, Chicago, IL, USA).

## 3 Results

### 3.1 CMV distribution in salivary and liver of SD Rats after inoculation

**Figure 1:**
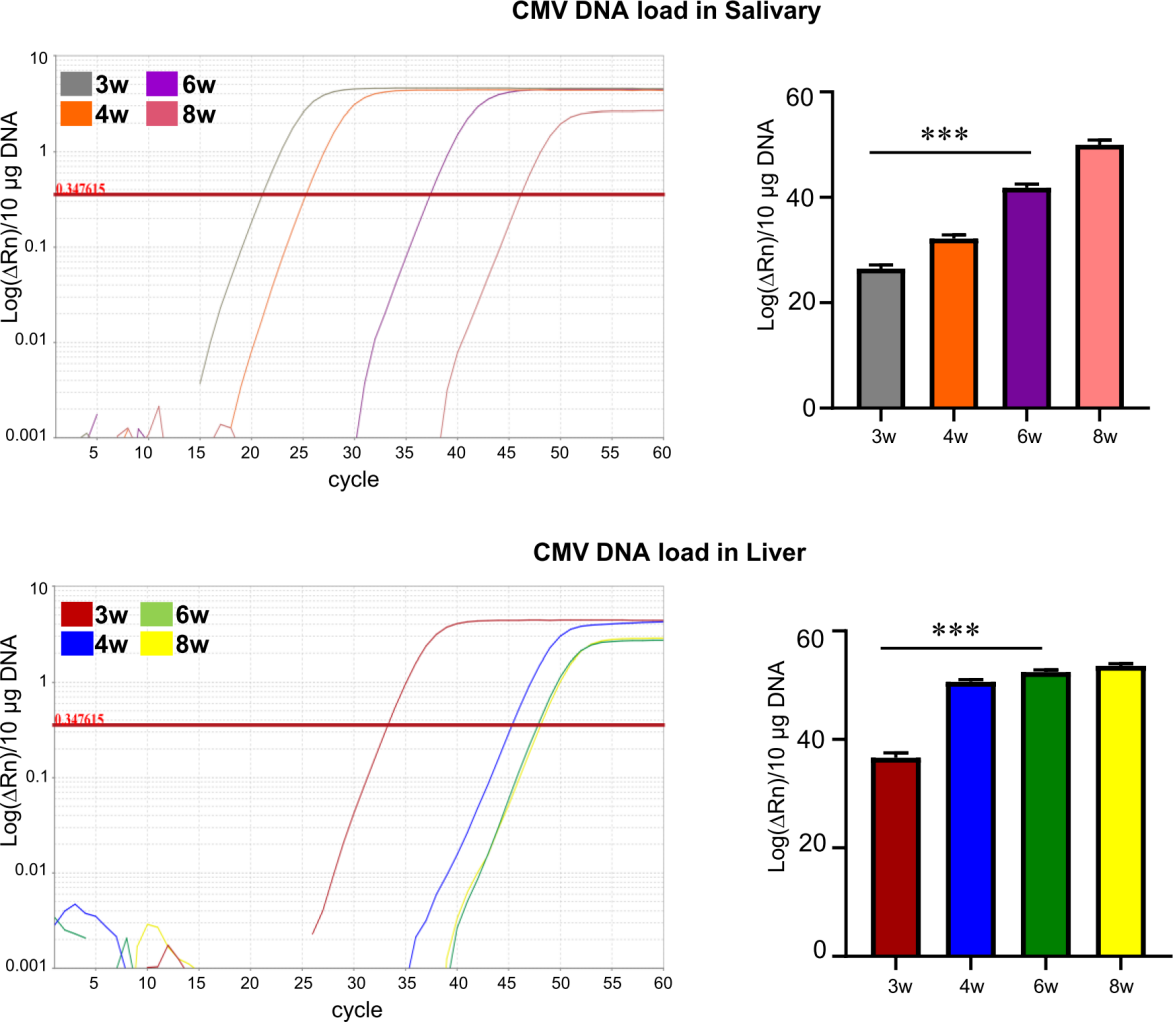
Presence of CMV in CMV infected SD rats. DNA loads were detected by quantitative PCR. Plots of log(ΔRn) vs.10 μg DNA from representative cases at 3w, 4w, 6w, 8w post-infection were shown. Data shown are representative of 3 independent experiments.

RCMV DNA presence was evaluated in the liver and salivary from infected rats. Significant reduction of viral DNA loads in both organs at 6w and 8w post-infection was observed as shown in **Figure 1**. The above findings suggested that peritoneal inoculation of CMV resulted in viral dissemination in the first 4 weeks. Furthermore, our previous studies by investigating the mRNA levels of *CMV IE1/2* and copy numbers of RCMV DNA indicated that a persistent low level of virus was only detected in the salivary glands while latency was established in organs such as liver at week 8 (Liu et al., 2022).

### 3.2 CD8^+^ T cell reconstitution after CMV infection

We previously found in CMV infected rats, the percentage of CD8^+^ T cells in splenocytes significantly increased at week 3 and declined to the level comparable with the control rats at week 6 (Liu et al., 2022). In the PBMCs of CMV-infected rats, similar alterations in T-cell constitution were identified (Liu et al., 2022).

According to our previous findings (Liu et al., 2022), examinations on the presence of CMV mRNA IE1/2, copy number of RCMV DNA and the systemic cytokine profiling as well as the T cell reconstitution indicated that primary infection of CMV led to systemic dissemination of virus to the internal organs and triggered the responsible immune responses during the first 4 weeks (peaked around week 3) in immuno-competent SD rats. After that, the immune status gradually recovered to the levels equivalent to the control rats, and CMV latency was established in organs such as liver. We confirmed that the CMV infection reached the peak at week 3, gradually declined and entered the latency at week 6 in the current study (**Figures 1**, **S1**).

### 3.3 Altered autophagic activity of CD8^+^ T cells during CMV infection

**Figure 2:**
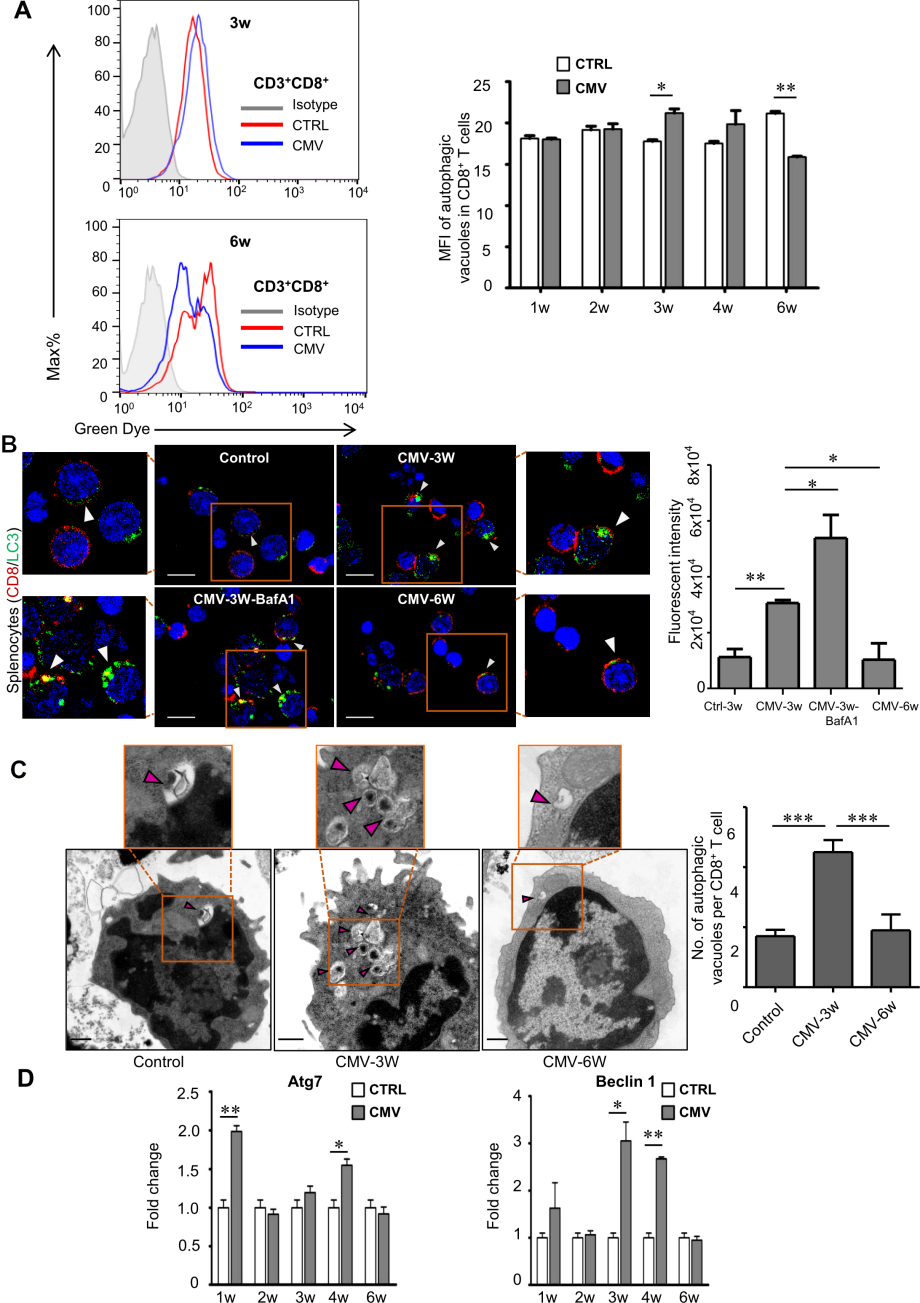
Dynamic changes of autophagy in CD8^+^ T cells after CMV infection. **(A)** Flow cytometry analyses revealed that autophagic activity of CD8^+^ T cells from spleen increased at week 3 and declined at week 6 after infection (n=3). **(B)** Splenocytes (CD8^+^ T cells) from rats infected for week 3 showed increased expression of autophagic markers LC3 compared with control and rats infected for 6 weeks. And this increase was further enhanced by autophagic inhibitor Bafilomycin A1 (n=3). Scale bars: 8 μm. **(C)** Augmented formation of autophagic vacuoles (arrowheads and inset) was visualized in CD8^+^ T cells of spleen in infected rats (week 3) under electron microscopy. The number of autophagic vacuoles was counted under the microscope from 10~15 representative cells for each group (n=3). Scale bars: 500 nm. **(D)** The mRNA levels of autophagic markers Atg7 and Beclin 1 in the splenocytes were up-regulated at early time points (week 1, 3, 4) through qRT-PCR and decreased to the control level at week 6 during CMV infection (n=3). MFI: mean fluorescence intensity; Data shown are representative of 3 independent experiments. Error bars indicate SEM; *p < 0.05, **p < 0.01, ***p < 0.001.

Since immunological functions of autophagy were suggested in adaptive immunity to virus infection (Xu et al., 2014), the autophagic activity of CD8^+^ T cells in splenocytes after CMV infection was evaluated. The results of flow cytometry showed that the fluorescence signals indicating autophagic vacuoles of CD8^+^ T cells were significantly increased (*p* < 0.05) at week 3 post CMV infection while decreased (*p* < 0.01) to the level lower than that of the control rats at week 6 (**Figures 2A**, **S2**). Consistently, CD8^+^ T cells (splenocytes) from CMV-infected rats at week 3 showed increased expression of LC3 (evaluated by the quantification of green fluorescence intensity across the entire CD8^+^ T cells with complete cell morphology) which was further enhanced by autophagy inhibitor Bafilomycin A1 (**Figures 2B**). Furthermore, examination under transmission electron microscopy demonstrated that there was enhanced formation of autophagosomes in CD8^+^ T cells of CMV-infected rats at week 3 while the number of autophagosomes decreased significantly at week 6 with blinded analysis (n=3, **Figure 2C**). Furthermore, we also found splenocytes from CMV-infected rats showed upregulated mRNA expressions of key autophagy molecules such as Atg7 and Beclin1 in the splenocytes at week 3 or 4 respectively after CMV infection by qRT-PCR (n=3, **Figure 2D**). This agreed with the dynamics of autophagy observed in CD8^+^ T cells.

### 3.4 Dynamic metabolic changes of CD8^+^ T cells during CMV infection and modification of metabolism by autophagy

**Figure 3:**
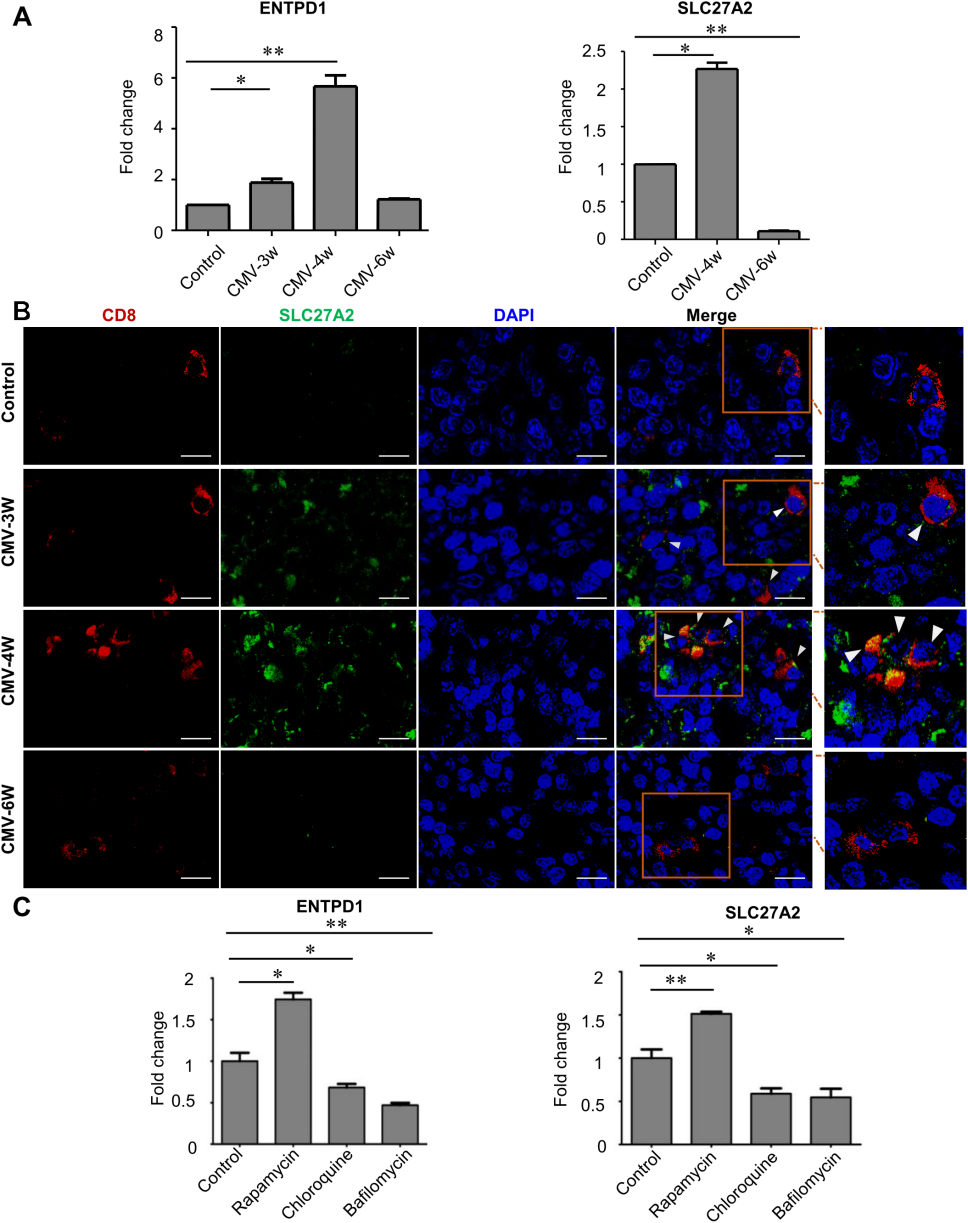
Dynamic alteration of metabolism in CD8^+^ T cells following CMV infection. **(A)** mRNA levels of ENTPD1 and SLC27A2 (metabolic markers) in splenocytes increased at early time points (week 3, 4) and declined at week 6 during CMV infection (n=3). **(B)** Immunofluorescent staining revealed that CD8^+^ T cell (red color) in infected rat spleen showed co-expression (merge, arrowheads) of SLC27A2 (green color) at 3w and 4w (n=3). Scale bars: 10 μm. Data shown are representative of 3 independent experiments. **(C)** Expression levels of ENTPD1 and SLC27A2 in splenocytes were modulated by autophagy enhancer (Rapamycin) and inhibitors (Chloroquine and Bafilomycin) *in vitro* (n=3). Error bars indicate SEM; *p < 0.05, **p < 0.01.

Metabolic changes in T cells after viral infection are essential for maintaining their functional integrity (Buck et al., 2015; Phan et al., 2016). Referring to the metabolism changes observed in human CMV infection (Hertoghs et al., 2010), the expression levels of two metabolic markers, ENTPD1 and SLC27A2 were evaluated in the splenocytes of infected rats at the time points in the current study (**Figure 3A**). The results revealed that the transcript levels of ectonucleoside triphosphate diphosphohydrolase 1 (ENTPD1; related to purinergic metabolism) were up-regulated at week 3 (*p* < 0.05) and week 4 (*p* < 0.01) after CMV infection and then declined to the level comparable to the control rats at week 6. Similarly, the expression level of solute carrier family 27 member 2 (SLC27A2; related to fatty acid metabolism) increased at week 4 (*p* < 0.05) while down-regulated at week 6 to the level **lower** than that of control rats (*p* < 0.01) (**Figure 3A**). Immunofluorescent double staining further confirmed that a parallel dynamic protein expression of SLC27A2 occurred in CD8^+^ T cells (increased at week 3, 4 while decreased at week 6 after CMV infection) (**Figure 3B**).

To further explore the association between autophagy and metabolism, we assessed the functional effects of autophagy enhancer (rapamycin) or inhibitors (Chloroquine and Bafilomycin) on the metabolism of rat splenocytes. We found that the transcript levels of ENTPD1 (*p* < 0.05) and SLC27A2 (*p* < 0.01) were augmented by autophagy inducer rapamycin (0.1 ng/µl). Meanwhile, the levels of ENTPD1 (*p* < 0.05, *p* < 0.01) and SLC27A2 (*p* < 0.05, *p* < 0.05) were down-regulated by autophagy inhibitors chloroquine (30 µM) and bafilomycin (100 nM) (**Figure 3C**). Collectively, these findings indicated that the metabolic alteration of CD8^+^ T cells in CMV infected rats might be regulated by autophagic pathway.

### 3.5 Dynamic expression of exhaustion markers in splenocytes during CMV infection

**Figure 4:**
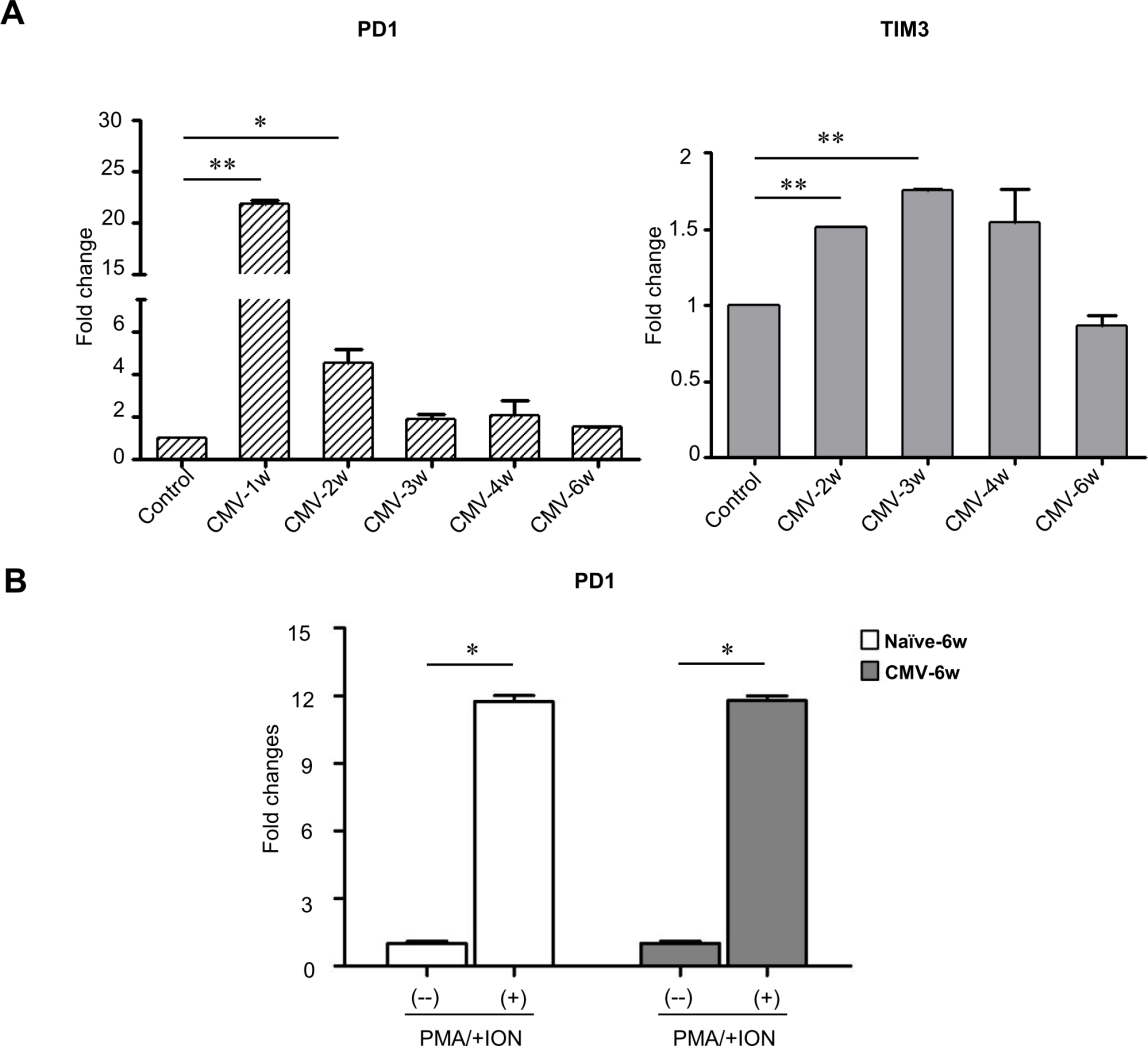
Expression of exhaustion markers in splenocytes after CMV infection. **(A)** The expression of PD1 and TIM3 (exhaustion markers) in the splenocytes increased at the early time points (1w, 2w or 3w) in the infected rats while became comparable to that in the control at week 6 (n=3). **(B)** PMA/Ionomycin induced equivalent up-regulation of PD1 expression in splenocytes from CMV latent or naive rats (n=3). Error bars indicate SEM; *p<0.05, **p<0.01.

T cells against persistent CMV infection show no exhaustion phenotype (Hertoghs et al., 2010). Here to explore whether the metabolic changes observed in CD8^+^ T cells of CMV-infected rats impact their functional efficacy, the expression levels of exhaustion markers programmed cell death protein 1 (PD1) and T cell immunoglobulin and mucin domain-3 (TIM3) in the splenocytes at different time points were evaluated. We found that the transcript levels of both PD1 and TIM3 were significantly up-regulated at early time points (week 1, 2; or week 2, 3) after primary infection but decreased to the level equivalent to the non-infected control at week 6 (**Figure 4A**). Furthermore, stimulation assay with phorbol 12-myristate 13-acetate/Ionomycin (PMA/ION) showed that PD1 levels in splenocytes of CMV-infected rats at latent phase (week 6) was augmented to the same degree as non-infected counterparts (**Figure 4B**). Our recent study also reported that the IFNγ production in CD8^+^ T cells isolated from splenocytes of CMV-infected rats at week 6 could be obviously increased after re-stimulation with CMV antigen pool (Liu et al., 2023), which is consistent with the findings. These results indicated that the splenocytes from CMV-infected rats were not functionally exhausted even though the metabolism changes took place in these cells.

## 4 Discussion

Priscott strain of RCMV shows significant biological difference compared with Maastricht strain and ALL-03 strain (Beisser et al., 1998; Loh et al., 2003; Voigt et al., 2005). The feasibility of inoculating Priscott strain in immuno-competent SD rats has been reported by previous studies in which virus was inoculated right after induction of carotid artery balloon injury in adult rats (400~450g) (Zhou et al., 1999). In comparison, our model with Priscott strain added values to RCMV-SD model in two aspects. Firstly, we identified the interactions, especially cellular immune responses between RCMV and SD rats under natural conditions (no other injury introduced except virus injection) for the first time. The observed significant increase of CD8^+^ T cells during acute CMV infection and a relativedrp predominance of CD4^+^ T cells in latent infection revealed host responses similar to those in human (Sester et al., 2002). Secondly, the model we set up covered a broader range of rat age, from week 3 (start of virus inoculation, ~100g) to week 11 (the end of monitoring, ~520g) old. Based on plaque assay and immunity monitoring in our previous results, we clarified that liver-specific latency in SD rats may develop as early as 6 weeks after viral inoculation (Liu et al., 2022). From the perspective of studying the role of CMV latency in liver graft injury, these data indicated that surgical transplantation can be conducted 6 weeks after primary infection when the rats are still in the suitable size (300 ± 20g) for delicate surgical manipulation. We believe this relative short term, together with the distinctive immunity dynamics, will enhance the attractiveness of RCMV-SD rat platform in simulating human CMV-related diseases in both transplantation and immunology. One potential limitation of the model is that unlike in humans or mouse CMV models, the presence of virus and T cell immunity in rats remains mostly uncharacterized because antibody against RCMV antigen and CMV-specific markers for rat T cells are not yet available. Therefore, developing CMV-specific markers for rat T cells (memory T cells, effector T cells etc.) deserves further attention as this may greatly improve the value of this model in answering questions not easily addressed in clinical research.

Studies in the past several decades established that metabolic programs drives T cell fate and function (Puleston et al., 2014; Xu et al., 2014; Buck et al., 2015). At the same time, autophagy has emerged as a key process in modulating T cell metabolism in recent years (Hubbard et al., 2010; Jia and He, 2011; Kabat et al., 2016). Currently the context-dependent roles of autophagy in regulating T-cell metabolic activity after viral infection are still not fully characterized, and identifying key regulators bridging the cross-talk between autophagy and T cell metabolism is an area of intense research (Dowling and Macian, 2018). In this study, we demonstrated that increase of autophagy in T cells during acute CMV infection was accompanied with the enhancement of metabolism. Consistently, *in vitro* pharmacological interference of autophagy also resulted in alteration of metabolism. Our findings suggested that autophagy may up-regulate metabolism to ensure sufficient energy production in the acute phase and switch the metabolism to a lower level to maintain the functional integrity of T cells in latent phase. This is the first report to demonstrate a dynamic interplay between autophagy and metabolic adaptation in CD8^+^ T cells during rat CMV infection. It may open the new perspective to understand the role of T cells in CMV-related diseases. It is worth noting that although the chemical drugs (rapamycin, chloroquine and bafilomycin) are widely applied to monitor autophagy in different disease models, they are non-specific activator or inhibitors (2010; Gupta et al., 2014). In this study, the consistent inhibitory effect with inhibitors (chloroquine and bafilomycin) which block autophagy via different mechanisms minimized the potential bias resulting from non-specific effects of these chemical drugs. Additionally, these findings are warranted to be confirmed with more specific manipulation of autophagy (eg, RNAi against the ATG genes) in future studies.

The immune status of CMV latency may provide the basis to investigate the mechanism of adverse effects related to CMV post liver transplantation. Among the metabolic markers we screened, markers relating to purinergic signaling (ENTPD1) and fatty acid metabolism (SLC27A2) demonstrated significant dynamic changes during CMV infection. ENTPD1, or CD39, the prototypic member of the ENTPDase family which degrades extracellular adenosine triphosphate (ATP) into adenosine monophosphate (AMP), plays important roles in the regulation of purinergic signaling in immune cells (Junger, 2011; Takenaka et al., 2016). The clinical analysis demonstrated that CMV infection enhanced the rejection post liver transplantation (Razonable and Humar, 2013). The previous research verified that the autophagy was critical regulator of memory CD8^+^ T cell formation in CMV infection (Puleston et al., 2014). It was reported that ENTPD1 contributed to the survival of T memory cells (Takenaka et al., 2016). How autophagy-ENTPD1 signaling impacted the function of CMV-specific memory CD8^+^ T cells and further influenced the increased rejection post liver transplantation deserved thorough investigation in the future study. Recent studies also indicated that ENTPD1 influenced cellular metabolism and mTOR-dependent autophagy by modifying purinergic signaling (Sun et al., 2013). Our observation that dynamic expression of ENTPD1 in the splenocytes during CMV infection paralleled the level of autophagy corroborated the findings as noted above. SLC27A2, also known as fatty acid transport protein 2 (FATP2), belongs to the fatty acid transport protein family (FATPs/solute carrier family 27) (Uchiyama et al., 1996; Steinberg et al., 1999; Tao et al., 2007). This protein converts free long-chain fatty acids into fatty acyl-CoA esters, and thereby is involved in the regulation of lipid biosynthesis and fatty acid degradation. So far there is a paucity of data regarding the function of SLC27A2 in viral immunity. Our recent study found the worse liver function and fibrosis in CMV latent patients post LDLT (Liu et al., 2022). The increase of SLC27A2 could activate inflammasome, resulting in the inflammation cascades (Liu et al., 2023). The previous upregulation of SLC27A2 in CMV-specific CD8^+^ T cells might be more easily enhanced by the stimulation of CMV reinfection or ischemia reperfusion injury during liver transplantation, leading to the severer inflammation and long-term fibrosis. Further investigations to determine the role of SLC27A2 in CMV latency and inflammation induction in liver transplantation are greatly warranted in future research.

In this study, we found the expression of PD1 and Tim3 in T cells decreased to control level during CMV latency and can be re-stimulated by PMA/Ionomycin (PMA/ION). This indicated that T cells in latent phase didn’t show an exhausted phenotype, which was consistent with CMV-specific T cells in humans. Additionally, we observed that T cells during latency showed similar viability as control T cells. Collectively, these findings suggested that the decreased metabolism of T cells during latency was an adapted change to internal environment rather than metabolism impairment. And autophagy may function as one of the major regulators to control this adaptation.

We acknowledge, however, that the *in vivo* data presented herein demonstrate a robust correlation between autophagy induction and metabolic reprogramming, and that pharmacological modulation of autophagy carries inherent risks of off-target effects. Follow-up investigations employing targeted knock-in or knock-out/down approaches are warranted to unequivocally establish the causal role of autophagy in CMV-driven metabolic alterations.

In summary, immunocompetent SD rats infected with the Priscott strain of rat cytomegalovirus exhibited cellular immune responses closely mirroring those observed in humans. Establishing this system provides an ideal preclinical platform for investigating the impact of CMV latency on clinical scenarios, such as liver transplantation. Furthermore, we observed a dynamic, parallel regulation of autophagy and metabolic markers in CD8^+^ T cells during CMV infection, which can be pharmacologically modulated *in vitro*. This novel finding implied that the impact of autophagy on T cell immunity needs to be taken into consideration when therapeutic strategy against CMV reactivation is developed for immuno-compromised patients.

## Data Availability

The raw data supporting the conclusions of this article will be made available by the authors, without undue reservation.

## Supplementary material

Supplementary Figure 1 The dynamic immune process of CMV infection in rats. CMV inoculation triggered a wave of immune response at early time points (week 1~4) after which organ specific viral latency ensued (week 6).

Supplementary Figure 2 The gating strategies of CD8^+^ T cells and autophagic vacuoles.

## References

[B1] Beisser P. S. Kaptein S. J. Beuken E. Bruggeman C. A. Vink C. (1998). The Maastricht strain and England strain of rat cytomegalovirus represent different betaherpesvirus species rather than strains. Virology 246, 341–351. doi: 10.1006/viro.1998.9196, PMID: 9657952

[B2] Bruggeman C. Debie W. Grauls G. Majoor G. Van Boven C. (1983). Infection of laboratory rats with a new cytomegalo-like virus. Arch. Virol. 76, 189–199. doi: 10.1007/BF01311103, PMID: 6307225

[B3] Bruggeman C. Meijer H. Bosman F. Van Boven C. (1985). Biology of rat cytomegalovirus infection. Intervirology 24, 1–9. doi: 10.1159/000149612, PMID: 2995270

[B4] Buck M. D. O’Sullivan D. Pearce E. L. (2015). T cell metabolism drives immunity. J. Exp. Med. 212, 1345–1360. doi: 10.1084/jem.20151159, PMID: 26261266 PMC4548052

[B5] Casazza J. P. Betts M. R. Price D. A. Precopio M. L. Ruff L. E. Brenchley J. M. . (2006). Acquisition of direct antiviral effector functions by CMV-specific CD4+ T lymphocytes with cellular maturation. J. Exp. Med. 203, 2865–2877. doi: 10.1084/jem.20052246, PMID: 17158960 PMC2118179

[B6] Castón J. J. Cantisán S. Gonzalez-Gasca F. Páez-Vega A. Abdel-Hadi H. Illescas S. . (2016). Interferon-γ production by CMV-specific CD8+ T lymphocytes provides protection against cytomegalovirus reactivation in critically ill patients. Intensive Care Med. 42, 46–53. doi: 10.1007/s00134-015-4077-6, PMID: 26537489

[B7] Chang C.-H. Pearce E. L. (2016). Emerging concepts of T cell metabolism as a target of immunotherapy. Nat. Immunol. 17, 364–368. doi: 10.1038/ni.3415, PMID: 27002844 PMC4990080

[B8] Dowling S. D. Macian F. (2018). Autophagy and T cell metabolism. Cancer letters. 419, 20–26. doi: 10.1016/j.canlet.2018.01.033, PMID: 29339212 PMC5937942

[B9] Ettinger J. Geyer H. Nitsche A. Zimmermann A. Brune W. Sandford G. R. . (2012). Complete genome sequence of the English isolate of rat cytomegalovirus (Murid herpesvirus 8). J. Virol. 86, 13838–13838. doi: 10.1128/JVI.02614-12, PMID: 23166247 PMC3503139

[B10] Fisicaro P. Barili V. Montanini B. Acerbi G. Ferracin M. Guerrieri F. . (2017). Targeting mitochondrial dysfunction can restore antiviral activity of exhausted HBV-specific CD8 T cells in chronic hepatitis B. Nat. Med. 23, 327–336. doi: 10.1038/nm.4275, PMID: 28165481

[B11] Gupta N. A. Kolachala V. L. Jiang R. Abramowsky C. Shenoi A. Kosters A. . (2014). Mitigation of autophagy ameliorates hepatocellular damage following ischemia-reperfusion injury in murine steatotic liver. Am. J. Physiol. Gastrointest Liver Physiol. 307, G1088–G1099. doi: 10.1152/ajpgi.00210.2014, PMID: 25258410 PMC4254956

[B12] Hertoghs K. M. Moerland P. D. van Stijn A. Remmerswaal E. B. Yong S. L. van de Berg P. J. . (2010). Molecular profiling of cytomegalovirus-induced human CD8+ T cell differentiation. J. Clin. Invest. 120, 4077–4090. doi: 10.1172/JCI42758, PMID: 20921622 PMC2964975

[B13] Hooth M.J. Nyska A. Bristol D.W. Bucher J.R. Chhabra R.S. Collins B.J. . (2010). Toxicology and carcinogenesis studies of 3, 3’, 4, 4’-tetrachloroazobenzene (TCAB) (CAS No. 14047-09-7) in Harlan Sprague-Dawley rats and B6C3F1 mice (gavage studies). Natl. Toxicol. Program Tech Rep. Ser. 558), 1–206., PMID: 21383777

[B14] Hubbard V. M. Valdor R. Patel B. Singh R. Cuervo A. M. Macian F. (2010). Macroautophagy regulates energy metabolism during effector T cell activation. J. Immunol., 185, 7349–57. doi: 10.4049/jimmunol.1000576, PMID: 21059894 PMC3046774

[B15] Jia W. He Y.-W. (2011). Temporal regulation of intracellular organelle homeostasis in T lymphocytes by autophagy. J. Immunol., 186, 5313–22. doi: 10.4049/jimmunol.1002404, PMID: 21421856

[B16] Junger W. G. (2011). Immune cell regulation by autocrine purinergic signalling. Nat. Rev. Immunol. 11, 201–212. doi: 10.1038/nri2938, PMID: 21331080 PMC4209705

[B17] Kabat A. M. Harrison O. J. Riffelmacher T. Moghaddam A. E. Pearson C. F. Laing A. . (2016). The autophagy gene Atg16l1 differentially regulates Treg and TH2 cells to control intestinal inflammation. Elife 5, e12444. doi: 10.7554/eLife.12444.025, PMID: 26910010 PMC4798959

[B18] Klenerman P. Oxenius A. (2016). T cell responses to cytomegalovirus. Nat. Rev. Immunol. 16, 367–377. doi: 10.1038/nri.2016.38, PMID: 27108521

[B19] Lemström K. Sihvola R. Bruggeman C. Häyry P. Koskinen P. (1997). Cytomegalovirus infection–enhanced cardiac allograft vasculopathy is abolished by DHPG prophylaxis in the rat. Circulation 95, 2614–2616. doi: 10.1161/01.cir.95.12.2614, PMID: 9193428

[B20] Ling C. C. Ng K. T. Shao Y. Geng W. Xiao J. W. Liu H. . (2014). Post-transplant endothelial progenitor cell mobilization via CXCL10/CXCR3 signaling promotes liver tumor growth. J. Hepatol. 60, 103–109. doi: 10.1016/j.jhep.2013.08.017, PMID: 23994383

[B21] Liu H. Yeung W. H. O. Pang L. Liu J. Liu X. B. Ng K. T. P. . (2023). Arachidonic acid activates NLRP3 inflammasome in MDSCs via FATP2 to promote post-transplant tumour recurrence in steatotic liver grafts. JHEP Rep. 5, 100895. doi: 10.1016/j.jhepr.2023.100895, PMID: 37916155 PMC10616418

[B22] Liu X. B. Liu H. Liu J. Cheung A. K. L. Zheng M. Z. Cheng J. L. . (2022). Cytomegalovirus latency exacerbated small-for-size liver graft injury through activation of CCL19/CCR7 in hepatic stellate cells. Transplantation 106, 519–530. doi: 10.1097/TP.0000000000003846, PMID: 34156186

[B23] Loh H. Mohd-Azmi M. Lai K. Sheikh-Omar A. Zamri-Saad M. (2003). Characterization of a novel rat cytomegalovirus (RCMV) infecting placenta-uterus of Rattus rattus diardii. Arch. Virol. 148, 2353–2367. doi: 10.1007/s00705-003-0173-y, PMID: 14648291

[B24] Loi M. Müller A. Steinbach K. Niven J. da Silva R. B. Paul P. . (2016). Macroautophagy proteins control MHC class I levels on dendritic cells and shape anti-viral CD8+ T cell responses. Cell Rep. 15, 1076–1087. doi: 10.1016/j.celrep.2016.04.002, PMID: 27117419

[B25] Man K. Ng K. T. Xu A. Cheng Q. Lo C. M. Xiao J. W. . (2010). Suppression of liver tumor growth and metastasis by adiponectin in nude mice through inhibition of tumor angiogenesis and downregulation of Rho kinase/IFN-inducible protein 10/matrix metalloproteinase 9 signaling. Clin. Cancer Res. 16, 967–977. doi: 10.1158/1078-0432.CCR-09-1487[pii, PMID: 20103676

[B26] Phan A. T. Doedens A. L. Palazon A. Tyrakis P. A. Cheung K. P. Johnson R. S. . (2016). Constitutive glycolytic metabolism supports CD8+ T cell effector memory differentiation during viral infection. Immunity 45, 1024–1037. doi: 10.1016/j.immuni.2016.10.017, PMID: 27836431 PMC5130099

[B27] Pickering H. Sen S. Arakawa-Hoyt J. Ishiyama K. Sun Y. Parmar R. . (2021). NK and CD8+ T cell phenotypes predict onset and control of CMV viremia after kidney transplant. JCI Insight 6, e153175. doi: 10.1172/jci.insight.153175, PMID: 34609965 PMC8663544

[B28] Puleston D. J. Zhang H. Powell T. J. Lipina E. Sims S. Panse I. . (2014). Autophagy is a critical regulator of memory CD8+ T cell formation. Elife 3, e03706. doi: 10.7554/eLife.03706.017, PMID: 25385531 PMC4225493

[B29] Razonable R. R. Humar A. (2013). Cytomegalovirus in solid organ transplantation. Am. J. Transplant. 13, 93–106. doi: 10.1111/ajt.12103, PMID: 23465003

[B30] Sester M. Sester U. Gärtner B. C. Girndt M. Meyerhans A. Köhler H. (2002). Dominance of virus-specific CD8 T cells in human primary cytomegalovirus infection. J. Am. Soc. Nephrol. 13, 2577–2584. doi: 10.1097/01.ASN.0000030141.41726.52, PMID: 12239248

[B31] Span A. Grauls G. Bosman F. Van Boven C. Bruggeman C. (1992). Cytomegalovirus infection induces vascular injury in the rat. Atherosclerosis 93, 41–52. doi: 10.1016/0021-9150(92)90198-P, PMID: 1317707

[B32] Stals F. S. Wagenaar S. S. Bruggeman C. A. (1994). Generalized cytomegalovirus (CMV) infection and CMV-induced pneumonitis in the rat: combined effect of 9-(1, 3-dihydroxy-2-propoxymethyl) guanine and specific antibody treatment. Antiviral Res. 25, 147–160. doi: 10.1016/0166-3542(94)90103-1, PMID: 7847876

[B33] Steinberg S. J. Wang S. J. Kim D. G. Mihalik S. J. Watkins P. A. (1999). Human very-long-chain acyl-CoA synthetase: cloning, topography, and relevance to branched-chain fatty acid metabolism. Biochem. Biophys. Res. Commun. 257, 615–621. doi: 10.1006/bbrc.1999.0510, PMID: 10198260

[B34] Sun X. Han L. Seth P. Bian S. Li L. Csizmadia E. . (2013). Disordered purinergic signaling and abnormal cellular metabolism are associated with development of liver cancer in Cd39/ENTPD1 null mice. Hepatology 57, 205–216. doi: 10.1002/hep.25989, PMID: 22859060 PMC3505255

[B35] Takenaka M. C. Robson S. Quintana F. J. (2016). Regulation of the T cell response by CD39. Trends Immunol. 37, 427–439. doi: 10.1016/j.it.2016.04.009, PMID: 27236363 PMC5215082

[B36] Tao W. Chang L. Yuan-Zhu X. Chang-Yan D. Bo Z. Hong-Tao X. . (2007). Isolation and cloning of porcine SLC27A2 gene and detection of its polymorphism associated with growth and carcass traits. Asian Australas. J. Anim. Sci. 20, 1169. doi: 10.5713/ajas.2007.1169

[B37] Uchiyama A. Aoyama T. Kamijo K. Uchida Y. Kondo N. Orii T. . (1996). Molecular cloning of cDNA encoding rat very long-chain acyl-CoA synthetase. J. Biol. Chem. 271, 30360–30365. doi: 10.1074/jbc.271.48.30360, PMID: 8939997

[B38] Vink C. Beuken E. Bruggeman C. A. (2000). Complete DNA sequence of the rat cytomegalovirus genome. J. Virol. 74, 7656–7665. doi: 10.1128/JVI.74.16.7656-7665.2000, PMID: 10906222 PMC112289

[B39] Voigt S. Sandford G. R. Hayward G. S. Burns W. H. (2005). The English strain of rat cytomegalovirus (CMV) contains a novel captured CD200 (vOX2) gene and a spliced CC chemokine upstream from the major immediate-early region: further evidence for a separate evolutionary lineage from that of rat CMV Maastricht. J. Gen. Virol. 86, 263–274. doi: 10.1099/vir.0.80539-0, PMID: 15659745

[B40] Weiss M. L. Anderson C. Medicetty S. Seshareddy K. B. Weiss R. J. VanderWerff I. . (2008). Immune properties of human umbilical cord Wharton’s jelly-derived cells. Stem Cells 26, 2865–2874. doi: 10.1634/stemcells.2007-1028, PMID: 18703664

[B41] Xu X. Araki K. Li S. Han J. H. Ye L. Tan W. G. . (2014). Autophagy is essential for effector CD8(+) T cell survival and memory formation. Nat. Immunol. 15, 1152–1161. doi: 10.1038/ni.3025, PMID: 25362489 PMC4232981

[B42] Yeung O. W. Lo C.-M. Ling C.-C. Qi X. Geng W. Li C.-X. . (2015). Alternatively activated (M2) macrophages promote tumour growth and invasiveness in hepatocellular carcinoma. J. Hepatol. 62, 607–616. doi: 10.1016/j.jhep.2014.10.029, PMID: 25450711

[B43] Zhou Y. F. Shou M. Guetta E. Guzman R. Unger E. F. Yu Z. X. . (1999). Cytomegalovirus infection of rats increases the neointimal response to vascular injury without consistent evidence of direct infection of the vascular wall. Circulation 100, 1569–1575. doi: 10.1161/01.CIR.100.14.1569, PMID: 10510062

[B44] Zhou Y. F. Shou M. Harrell R. F. Yu Z. X. Unger E. F. Epstein S. E. (2000). Chronic non-vascular cytomegalovirus infection: effects on the neointimal response to experimental vascular injury. Cardiovasc. Res. 45, 1019–1025. doi: 10.1016/S0008-6363(99)00394-6, PMID: 10728428

[B45] Zhu J. Shearer G. M. Marincola F. M. Norman J. E. Rott D. Zou J. P. . (2001). Discordant cellular and humoral immune responses to cytomegalovirus infection in healthy blood donors: existence of a Th1-type dominant response. Int. Immunol. 13, 785–790. doi: 10.1093/intimm/13.6.785, PMID: 11369706

